# A reference relative time-scale as an alternative to chronological age for cohorts with long follow-up

**DOI:** 10.1186/s12982-015-0043-6

**Published:** 2015-12-18

**Authors:** Margaret Anne Hurley

**Affiliations:** College of Health and Wellbeing, University of Central Lancashire, Preston, PR1 2HE UK

**Keywords:** Operational failure time, Life expectancy, Time transformation, Age-at-risk, Cumulative hazard, Attained age

## Abstract

**Background:**

Epidemiologists have debated the appropriate time-scale for cohort survival studies; chronological age or time-on-study being two such time-scales. Importantly, assessment of risk factors may depend on the choice of time-scale. Recently, chronological or attained age has gained support but a case can be made for a ‘reference relative time-scale’ as an alternative which circumvents difficulties that arise with this and other scales. The reference relative time of an individual participant is the integral of a reference population hazard function between time of entry and time of exit of the individual. The objective here is to describe the reference relative time-scale, illustrate its use, make comparison with attained age by simulation and explain its relationship to modern and traditional epidemiologic methods.

**Results:**

A comparison was made between two models; a stratified Cox model with age as the time-scale versus an un-stratified Cox model using the reference relative time-scale. The illustrative comparison used a UK cohort of cotton workers, with differing ages at entry to the study, with accrual over a time period and with long follow-up. Additionally, exponential and Weibull models were fitted since the reference relative time-scale analysis need not be restricted to the Cox model. A simulation study showed that analysis using the reference relative time-scale and analysis using chronological age had very similar power to detect a significant risk factor and both were equally unbiased. Further, the analysis using the reference relative time-scale supported fully-parametric survival modelling and allowed percentile predictions and mortality curves to be constructed.

**Conclusions:**

The reference relative time-scale was a viable alternative to chronological age, led to simplification of the modelling process and possessed the defined features of a good time-scale as defined in reliability theory. The reference relative time-scale has several interpretations and provides a unifying concept that links contemporary approaches in survival and reliability analysis to the traditional epidemiologic methods of Poisson regression and standardised mortality ratios. The community of practitioners has not previously made this connection.

## Background

In recent years, epidemiologists have debated which of the several possible time-scales to use for survival analysis for longitudinal studies and it has been argued that chronological age as the time-scale is preferable to the traditional time-on-study [1, 2]. The utility of chronological age has been disputed because, it is argued, that without further covariate adjustment for age at entry to the cohort or without ‘left-truncation’ the unadjusted age scale is inferior in performance to other models [3]. When adjustment is made for age at entry then, it has been suggested, model coefficients for risk factors of interest differ little between models using the chronological age scale and those using time-on-study [3, 4]. However, others have reported substantial differences in the assessment of risk factors depending on the time-scale used for the analysis [5–7]. The choice of correct time-scale is important because the substantive findings of large cohort studies might be called into question if it were believed that an inappropriate choice leads to seriously biased estimates of hazard ratios for risk factors [8]. What is clear is that considerable care is required in the choice of time-scale for any particular application; one example being when an environmental exposure is highly correlated with the calendar time-scale [9]. The use of alternative time-scales has been discussed widely in the context of reliability and performance and the definition of a good time-scale has been proposed [10].

Although there is still no general consensus, chronological age as time-scale has gained moderate acceptance for the analysis of survival data from cohort studies. With chronological age as the time-scale, age is usually deemed to be truncated on the left at age of entry to the study. Cox regression is the modelling framework of choice to assess risk factors and the baseline hazard is a nonparametric function of age. This assumption is problematic for long running studies which accrue participants over a period of time since the hazard cannot be a function of age alone but must also depend on calendar time. For example, the mortality rate of a white male of 60 years in California in 1984 would not be the same as that of a white male of 60 years in California in 2004 since longevity would have improved. Using chronological age leaves unresolved the issue of calendar time when participants enter the cohort at different calendar times and this issue is not trivial for studies which span several decades. Cohorts and particularly occupational cohorts, often have these features, that participants join the cohort at different ages and at different calendar times and are followed-up for long periods of time thereafter. One recommendation is to stratify on birth cohort in the Cox regression model using 5 or 10 yearly intervals but, for long running cohorts, this can result in very many strata [1, 3], although strata of varying lengths can be used to reduce the total number. Another possibility is to include age at entry as a covariate but modelling age-at-entry may require a complex sub-model to make adequate adjustment [5]. Stratification or covariate adjustment is not the only answer and a viable alternative, which has received little attention, is to take a relative survival approach to the analysis of cohorts with long follow-up. The aim of this paper is to publicise this alternative approach and to indicate how this addresses the issue of accommodating age at entry, birth cohort, differing entry times and changing longevity.

Relative survival has a long history in epidemiology [11–13]. Relative survival compares the mortality (or other event of interest) in a study population to that in an appropriate reference population. The standardised mortality ratio (SMR) is a primary traditional estimate of relative mortality and is defined as the number of events occurring in a study group relative to the number of events expected from the event rates observed in the reference population. The Poisson regression method for event counts, which uses expected number of events from the reference population in place of the person—years of observation, has been used widely in the past to model the association of risk factors with mortality or disease outcome [14–16]. Although not always recognised as such, the Poisson regression method is equivalent to modelling the SMR [17]. Poisson regression has lost popularity in recent years because it is now well recognised that modelling aggregated counts has less power to identify risk factors compared to modelling individual survival times using individual participant data (IPD). Although an extension of Poisson regression to model IPD has been described [18], Cox regression for individual survival times still comprises the regression method of first choice, since it makes no statistical distributional assumptions.

The objective of this communication is to compare, as an illustration, a standard analysis using chronological age as the time-scale, left-truncated at age of entry to the study, and with stratification for birth cohort in the Cox model with the analysis of individual survival times on a reference relative time-scale. The intention is to demonstrate that this approach provides a viable alternative to the current standard analysis and is an approach which circumvents much of the controversy surrounding the choice of time-scale. Also, it has appealing interpretations and is connected to the traditional methods of Poisson regression and SMR.

## Methods

This section described more fully the two time scales being compared; the new reference relative time-scale and the chronological age time-scale. Following this the illustrative cohort data is introduced and the statistical analyses of the data are described. The section finishes with a methodological description of the simulation study which was used to establish existence of any bias and the relative power of the two time-scales to detect a differential mortality risk between men and women of light smoking compared to non-smoking.

### The reference relative time-scale

An individual measure of relative survival has been described previously in which, each participant’s actual survival time is transformed to a new scale which is the expected residual cumulative distribution function from a reference population [19, 20]. This measure lies in the range 0.0 to 1.0. For example, an individual participant‘s measure of 0.80 implies that this participant’s survival time is longer than 80 % of their peers in the reference population where peers are defined as people of the same gender, date of birth and other key demographics such as race and location. Modelling of the risk factors is then performed in which the response variable is the transformed survival time, Y, and the regression method is the Cox model [20]. Cox regression is valid even though the response measure, Y, is bounded above by 1.0 and does not have the fundamental attribute of time, that of being unbounded above. If the measure Y is further transformed to the measure Z where Z = −ln(1−Y) then Z has the property that the Cox model fitted to the measure Z gives identical regression estimates and fit to the Cox model fitted to the measure Y. The measure Z is also bounded below by 0.0 but Z is not bounded above and so has the attributes of a realistic time-scale. The measure Z is the reference relative time.

More specifically, if an individual of gender g enters the study at age a_0_ at calendar time s_0_ and exits at age a_1_ at calendar time s_1_ then ${\text{Z}} = \int_{{s_{0} }}^{{s_{1} }} {\lambda \left( {g,a,s} \right)ds}$ where *λ*(*g, a, s*) is the hazard in the reference population for a person of gender g, at age a at calendar time s. The reference population may be defined by further demographics such as race and location, but the pertinent point is that the integral is taken over the hazard function for the individual participant’s peer group in the reference population. Thus the individual’s relative time is the cumulative hazard of the peer group in the reference population over the calendar time on study of the individual participant. The survival censoring indicator is the same as that for chronological age, which is whether the individual participant had an event at time s_1_ or was censored.

**Fig. 1:**
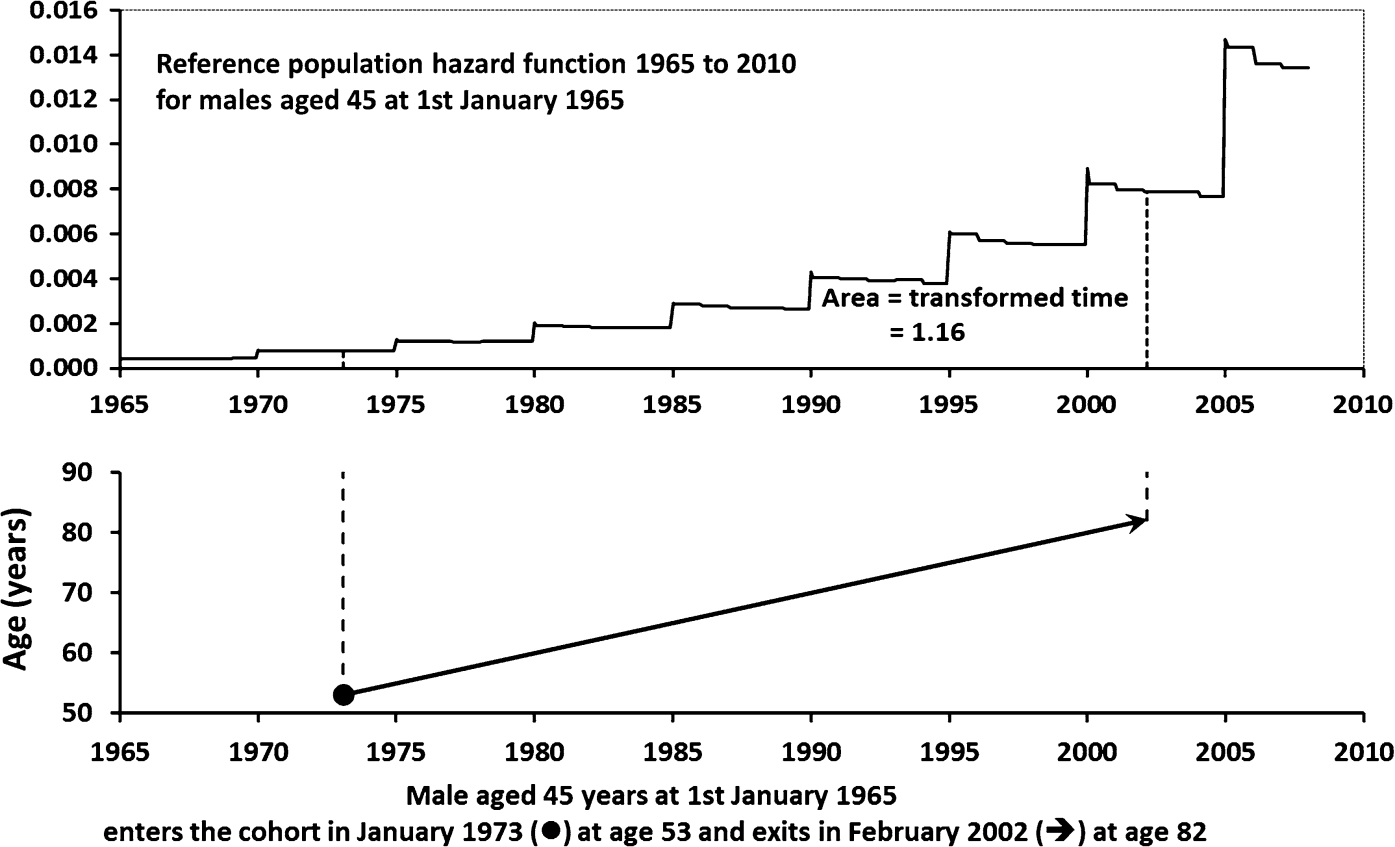
Graphical representation of the way in which reference relative time is computed as the integral under the hazard function (cumulative hazard) obtained from a reference population

The transformation, Z, transforms both censored and uncensored survival times to a new relative time-scale, called the reference relative time-scale. Figure 1 shows a representation of how the reference relative time-scale is obtained. The reference relative time-scale has a number of interpretations, some with an intuitive appeal and some which, at first sight, may seem counter-intuitive.

1. The reference relative time-scale is the expected number of events for the individual participant during their time-on-study. A reference relative survival time of 1.0 implies that the individual reached their residual life expectancy from entry based on the reference population. A reference relative survival greater than 1.0 implies that the individual had exceeded their residual life expectancy and a value less than 1.0 that they had died or embarked before reaching residual life expectancy.
2. The reference relative time-scale is a weighted version of the real time-on-study. Real time is ‘stretched’ when the hazard (force of mortality) in the reference population is high but time is ‘shrunk’ when the hazard is low. So a calendar time period such as 5 years lived by an older individual participant between 1990 and 1995, has a longer reference relative time value than 5 years between 1990 and 1995 lived through by a younger individual. Shrinking time is a form of accelerating time whereas stretching time, decelerates time.
3. The reference relative time-scale is the reciprocal of an individual participant’s own SMR (divided by 100 if the latter is expressed as a percentage). If the SMR is calculated for a study group of size one then the reference relative time would be the reciprocal of this value. Thus, an individual participant with an SMR of 50 % would have a reference relative survival time of 2.0. For this individual, the reference relative time on study was so long that the individual would have been ‘expected’ to have died twice, with an explanation in the following sense. On calendar time of entry the individual has an expected residual lifetime. If the individual achieves the end of this expected residual lifetime, they would then have a further (but much shorter) expected residual lifetime. If they achieve the end of this second residual lifetime then the reference relative survival time becomes 2.0. A log-linear model for the hazard on the reference relative time scale is therefore also a log-linear model for the individual participants’ SMRs.
4. The reference relative time-scale has a simple game-theoretic interpretation as the final score in a game. In each time period, the individual player joins battle against opponents that are intent on eliminating the individual. The number of opponents is proportional to the hazard in the reference population (force of opposition or mortality). If the individual eliminates all the opponents then the player banks points proportional to the number of opponents overcome. The cumulative number of banked points on exit from the game measures the individual’s total success during time-on-play in the game. Censoring applies to the banked points if on exit from the game the individual participant has not been eliminated. So, the reference relative time-scale is the cumulative amount of hazard that the individual has confronted before experiencing an event and is a measure of game success.

Modelling on the reference relative time-scale obtained from the transformation Z has two significant benefits. Firstly, as well as using the Cox regression, relative time can be modelled using the full range of parametric survivals models, such as exponential and Weibull models. Secondly, if risk factors and other variables are all categorical, then modelling reference relative time using an exponential distribution is equivalent to Poisson regression using observed and expected counts. If risk factors and other variables contain quantitative covariates then modelling reference relative times is an extension of Poisson regression to IPD. Thus modelling on the reference relative time-scale can be understood as a generalisation of the traditional Poisson regression method to IPD and to more complex survival distributions. Reference relative survival, therefore, provides a unifying conceptual framework which encompasses both traditional and contemporary methods of analysis.

The reference relative time-scale is an example of an alternative time-scale or ‘operational failure time-scale’ which has been discussed in the literature on reliability and performance of systems [10]. It meets the criteria for definition as an ‘ideal time-scale’ which is defined by four features (1) scientific relevance (2) parsimonious and accurate description of variation in failure times under different conditions (3) a compact statistical distribution on the transformed time-scale and (4) succinct and meaningful summarization of the effects of covariates of interest. An ‘ideal time-scale’ arises because the hazard in the reference population provides a time-varying external continuous covariate during each individual participant’s time-on-study.

### The chronological age time-scale

Currently, the conventional approach for survival analysis for cohort data with long follow-up, in which participants enter at different calendar times and where ages at entry are variable is to use the time-scale of chronological age with left truncation for age at entry. With this approach adjustment for birth cohort is made by using a stratified Cox model with 5 or 10 year intervals [3, 5] or intervals of varying size as appropriate.

### Illustrative cohort data

Analyses of a long running cohort of British cotton mill workers (1966–2007) have been previously described. The cohort was founded with the aim of understanding the long-term effects of exposure to environmental particulates on respiratory mortality and morbidity. The cohort has provided evidence that working with cotton reduced the risk of lung cancer death and that light smoking at baseline predicted higher mortality for women compared to men [21–23]. These results were obtained using the reference relative time-scale as the mode of analysis and the primary inferential topic of interest here was to demonstrate that these results were robust to the choice of time-scale.

Ages at entry to the cohort were in the range 15–81 years (quartiles, 32, 45 and 56 years) over the period 1966–1974 and participants’ year of birth was in the range 1885–1954. Smoking consumption was categorised at baseline as non-smoker, light (1–14), medium (15–24), heavy (25+ cigarettes per day) smoker and former smoker. The cohort contained both men and women mostly working full-time in all of the mill environments. Other variables measured at baseline included; the presence of the lung disease of cotton mill workers known as Byssinosis, the presence of cough and phlegm for at least 3 days per week for at least 3 months of the year, how long the worker had worked in the cotton industry and lung function expressed as forced expiratory volume in one second adjusted for age, gender and height (FEV_1_% predicted) and the ratio of FEV_1_% predicted to forced vital capacity (FVC). Participants alive at age 90 were censored. Table 1 illustrates the cohort data using 10 typical but hypothetical individual participants. Also, Table 1 shows the type of values produced by the transformation to reference relative survival time for the hypothetical individuals for cohort entry at one calendar time point. The participants with identifiers 1, 2 and 3 all had the same time on study but had different ages at entry. The first two participants of these three had small reference relative times because they entered at a young age, whereas the third participant entered at 40.8 years and their reference relative time on study was >1.057 showing that they had just attained their expected lifetime when they exited from the study. The participant with identifier 10 entered at age 60.7 years and their reference relative time on study was 1.756 which shows that they had well exceeded their expected residual lifetime from age at entry.

**Table 1:** Ten hypothetical participants entering the British Cotton Workers’ Cohort in December 1966 at first medical examination

ID	Age at entry (years)	Time on study (years)	Status at exit	Reference relative time	SMR (%)	Gender	Smoking status	Time worked in cotton (years)	FEV_1_% predicted^a^	FEV_1_% of FVC^b^	Cough^c^	Byssinosis^d^
1	25.8	41.2	Alive	>0.133	<751	M	N	7	91	76	A	A
2	28.7	41.2	Alive	>0.172	<581	F	N	6	101	78	A	A
3	40.8	41.2	Alive	>1.057	<95	M	M	21	93	77	A	A
4	45.7	22.6	Died	0.196	512	F	N	25	52	83	P	P
5	50.6	14.1	Died	0.229	437	F	N	33	107	87	A	A
6	51.6	24.7	Died	0.413	242	F	H	25	115	47	P	A
7	52.4	13.3	Died	0.248	403	M	L	30	101	59	A	A
8	55.2	31.3	Died	1.188	84	F	L	35	49	89	P	A
9	59.6	27.9	Died	1.355	74	F	F	41	63	68	A	P
10	60.7	29.3	Alive	>1.756	<57	F	N	43	74	81	P	A

These data are typical of participants in the study but do not correspond to any true participants *F* female, *M* male , *N* Never smoked, *L* 1–14 cigarettes per day, *M* 15–24 cigarettes per day, *H* 25 cigarettes or more per day, *F* former smoker

^a^Forced expiratory volume (FEV_1_) as a % of the normal FEV_1_ for a participant of this age, gender and height

^b^Forced expiratory volume (FEV_1_) as a % of forced vital capacity (FVC)

^c^Cough and phlegm at least 3 days per week for at least 3 months of the year: *A* absent, *P* present

^d^Lung disease of cotton workers, *A* Byssinosis absent, *P* Byssinosis present at grade ½ to 2

### Ethics approval

Ethical approval was obtained from the University of Central Lancashire’s Faculty of Health Research Ethics Committee which accepted that the study had been granted exemption by the Department of Health’s National Information Governance Board from the need to obtain informed consent from individuals retrospectively to participate in the mortality study. In addition the Medical Research Information service at the NHS Information Centre granted permission for the study to receive vital status data.

### Statistical analyses

To test the utility of the reference relative survival time-scale, two regression analyses were compared using the cotton mill workers’ cohort data. The first analysis was the conventional analysis that used age as the time-scale with left truncation in a stratified Cox model with 24 strata, representing 12 5-year birth cohorts per gender as suggested in the literature [1, 3]. The second was the analysis using the reference relative time-scale calculated using population mortality rates for England and Wales. The numbers of deaths by gender and by age in 5-year bands were obtained for each year from 1966 to 2007 and the corresponding mid-year population size estimates for England and Wales were also obtained [24]. The numbers of deaths were divided by the corresponding population size and then by 12 to obtain a monthly mortality hazard rate. The integral of the hazard rate was computed using a time interval of one month as an adequate approximation. Figure 1 gives an example of the estimated monthly mortality hazard for male aged 45 on 1 January 1965. The hazard is a step function because mortality was published in 5-year bands. The effect of improving longevity due to better health and social care is discernible in the declining hazard over a 5-year interval at the plateau on each step.

The analyses estimated the hazard ratio  (HR) for light, medium, heavy and former smoker relative to non-smoker together with HRs for other risk factors measured at baseline. The analyses also estimated the women to men relative risk ratios (RRR) for the four baseline categories of cigarette consumption; light, medium, heavy and former smoking. The relative risk ratio was defined by RRR = HR of female smokers to female non-smokers/HR of male smokers to male non-smokers where HR was the hazard ratio. The RRRs were the single degree of freedom components of the interaction between gender and consumption category and were estimated using indicator variables. Both analyses fitted the Cox model, but the Weibull and the exponential models were also fitted for the survival times on the reference relative time-scale. The analyses were performed in the R programming language and environment [25].

### Simulation study

In order to compare the bias and power of the reference relative time-scale to that of chronological age at risk time-scale a simulation study was carried out. Each simulation included all the participants from the illustrative cohort together with the participant’s age at entry, gender and date of entry to the study being kept fixed at their observed values. All the participant’s covariate and factor values were also kept fixed at their observed values. Each participant’s lifetime from date and age of entry was then simulated according to a model which calculated the participant’s hazard of dying in each calendar month following date of entry. For each month, in turn moving forward in time, a uniform random variable was generated. The first month, in the sequence, for which the random variable was less than the monthly hazard was selected as the simulated time of death. Once simulation of time of death was completed for all participants, a model was fitted by Cox regression using the reference relative time-scale and by a stratified Cox regression using the age at risk time-scale.

In the simulation, the monthly hazard for each participant was calculated from a combination of the model parameters and the England and Wales reference population monthly hazards. The model assumed that the hazard of dying was proportional to smoking status; non-smoker, light smoker, medium smoker, heavy smoker or former smoker with relative parameters α_0_, α_1_, α_2_, α_3_, α_4_ respectively and where α_0_ = 1. These parameters applied to both males and females except in the case of light smoking females for whom the relative parameter was βα_1_. In the simulation α_0_, α_1_, α_2_, α_3_, α_4_ were kept fixed at the values of 1.00, 1.24, 1.83, 2.08 and 0.93. Each simulation was for a fixed value of β in the range 1.0–1.4. The actual hazard used in each month was obtained from these model parameters and the numbers of participants in each gender and smoking category in the illustrative cohort. For males, if there were n_0_, n_1_, n_2_, n_3_, n_4_ in the categories; non-smoker, light smoker, medium smoker, heavy smoker and former smoker and the monthly hazard in the reference population was h_m_ then the hazard for non-smokers, h_0_ was computed by.

$${\text{h}}_{0} = {\text{ h}}_{\text{m}} \left( {{\text{n}}_{0} + {\text{ n}}_{ 1} + {\text{ n}}_{ 2} + {\text{ n}}_{ 3} + {\text{ n}}_{ 4} } \right)/\left( { \alpha_{0} {\text{n}}_{0} + \, \alpha_{ 1} {\text{n}}_{ 1} + \, \alpha_{ 2} {\text{n}}_{ 2} + \, \alpha_{ 3} {\text{n}}_{ 3} + \, \alpha_{ 4} {\text{n}}_{ 4} } \right)$$

The hazards for light smokers, medium smokers, heavy smokers and former smokers were given by α_1_h_0_, α_2_h_0_, α_3_h_0_ and α_4_h_0_ respectively. For females the calculation of the hazard was analogous except that α_1_ was replaced by βα_1_. This method of calculating the relative hazards had the effect that the average over a cohort with the observed mix of smoking grade would equal the reference population monthly hazard. This method generated realistic lifetimes with the appropriate relative hazards given by the model parameters. This allowed the two methods to be fairly compared in terms of their power to detect a value of β greater than 1.0 which would indicate a gender difference in the relative risk of light smoking and any bias in the estimation of β. It also allowed a check on the power of the null value of β = 1. For each value of β, 200 simulations were carried out and testing to estimate power used a 5 % significance level for the test.

The simulation also provided an opportunity to compare the methods when the reference population used to obtain the reference relative times was not an appropriate population for the cohort under study. This was achieved by increasing the monthly hazard of dying in the simulation by an additional multiplying factor which was an exponential function of age; exp (0.002 × age). This increased the hazard by about 4 % at age 20 and 13 % by age 60. This produced disproportionately shorter simulated lifetimes than in the reference population used to obtain the reference relative time-scale.

## Results

### Illustrative cohort data

A comparison of the fitted Cox model using the reference relative time-scale with the stratified Cox model using chronological age as the time-scale is presented in Table 2. Gender main effect was fixed at 1.0 in the stratified model due to the choice of stratification which included gender-specific strata. Previously, the proportional hazards assumption was tested for the Cox model using the reference relative time-scale by calculating the correlation between the Schoenfeld residuals and the transformed survival times and the assumption was shown to be satisfied [23]. Overall, there was a good measure of agreement between the two approaches in the parameter estimates and the 95 % confidence intervals for the risk factors of interest, suggesting that the two methods of analysis were comparable.

**Table 2:** Hazard ratios (HR) from the Cox regression model for all-cause mortality to 31 December 2007 using chronological age and reference relative time as the time-scales

	Chronological age as time-scale stratification by birth year and gender				Reference relative time as time-scale			
	Cox model				Cox model			
	HR	95 % CI		P value	HR	95 % CI		P value
Never smoked	1.00				1.00			
Light smoking	1.22	1.01	1.47	0.043	1.21	1.00	1.46	0.055
Medium smoking	1.65	1.35	2.01	<0.001	1.64	1.34	2.00	<0.001
Heavy smoking	1.96	1.46	2.62	<0.001	1.99	1.49	2.65	<0.001
Former smoking	0.90	0.66	1.22	0.481	0.89	0.66	1.22	0.475
Male	1.00				1.00			
Female	1.00				1.07	0.89	1.29	0.471
RRR light smoking	1.27	1.00	1.61	0.046	1.35	1.07	1.70	0.012
RRR medium smoking	1.06	0.82	1.38	0.657	1.15	0.89	1.49	0.290
RRR heavy smoking	0.96	0.59	1.54	0.853	1.00	0.63	1.61	0.990
RRR former smoking	1.07	0.66	1.75	0.774	1.10	0.68	1.78	0.705
Byssinosis absent	1.00				1.00			
Byssinosis present	1.01	0.90	1.12	0.902	1.04	0.93	1.15	0.535
Cough and phlegm absent	1.00				1.00			
Cough and phlegm present	1.10	1.00	1.21	0.061	1.07	0.97	1.18	0.155
One decade in the cotton industry	0.97	0.93	1.02	0.202	0.91	0.88	0.95	<0.001
FEV_1_: 10 % decrease below normal	1.08	1.05	1.11	<0.001	1.07	1.04	1.10	<0.001
FEV_1_ to FVC ratio: decrease of 10 %	1.04	0.98	1.10	0.186	1.01	0.96	1.07	0.726

The fitted model coefficients for two alternative fully parametric survival models, the Weibull and the exponential, when fitted to the reference relative times, are shown in Table 3. The Weibull was previously identified as a well-fitting parametric model for the reference relative survival times in the cotton mill workers’ study [23]. It is clear from the parameter estimates in Table 3 that the RRR for light smoking, 1.35 for the Weibull model, significantly exceeded the ‘no difference’ value of 1.0 and this indicates that the relative risk of light smoking compared to never having smoked was predicted at one-third greater for women than for men. Furthermore, a lung function at 10 % below the reference standard for a person of a given gender, age and height had a parameter value 1.07, indicating a significant predicted 7 % increase in mortality hazard. This increase was after taking into account the effect of smoking by including smoking and gender in the model.

**Table 3:** Hazard ratios (HR) from the Weibull and exponential regression models for all-cause mortality to 31^st^ December 2007 using reference relative time as the time-scale

	Relative time as time-scale				Relative time as time-scale			
	Weibull model*				Exponential model**			
	HR	95 % CI		P value	HR	95 % CI		P value
Never smoked	1.00				1.00			
Light smoking	1.21	1.00	1.46	0.051	1.20	0.99	1.45	0.058
Medium smoking	1.68	1.38	2.05	<0.001	1.63	1.34	1.99	<0.001
Heavy smoking	2.01	1.51	2.68	<0.001	1.96	1.47	2.61	<0.001
Former smoking	0.89	0.66	1.21	0.465	0.90	0.66	1.22	0.485
Male	1.00				1.00			
Female	1.10	0.92	1.32	0.304	1.08	0.90	1.30	0.383
RRR light smoking	1.35	1.07	1.70	0.012	1.33	1.05	1.68	0.016
RRR medium smoking	1.13	0.87	1.46	0.361	1.13	0.87	1.46	0.351
RRR heavy smoking	0.98	0.61	1.58	0.942	0.98	0.61	1.58	0.943
RRR former smoking	1.11	0.68	1.80	0.681	1.10	0.68	1.78	0.702
Byssinosis absent	1.00							
Byssinosis present	1.04	0.93	1.16	0.484	1.04	0.93	1.16	0.524
Cough and phlegm absent	1.00							
Cough and phlegm present	1.07	0.97	1.18	0.158	1.08	0.98	1.19	0.142
One decade in the cotton industry	0.91	0.88	0.94	<0.001	0.92	0.89	0.96	<0.001
FEV_1_: 10 % decrease below normal	1.07	1.04	1.10	<0.001	1.07	1.04	1.10	<0.001
FEV_1_ to FVC ratio: decrease of 10 %	1.01	0.95	1.07	0.763	1.02	0.96	1.0800	0.571

* LogL = −1813.7, shape 1.08 (95 % CI 1.04–1.12)

** LogL = −1822.9

**Fig. 2:**
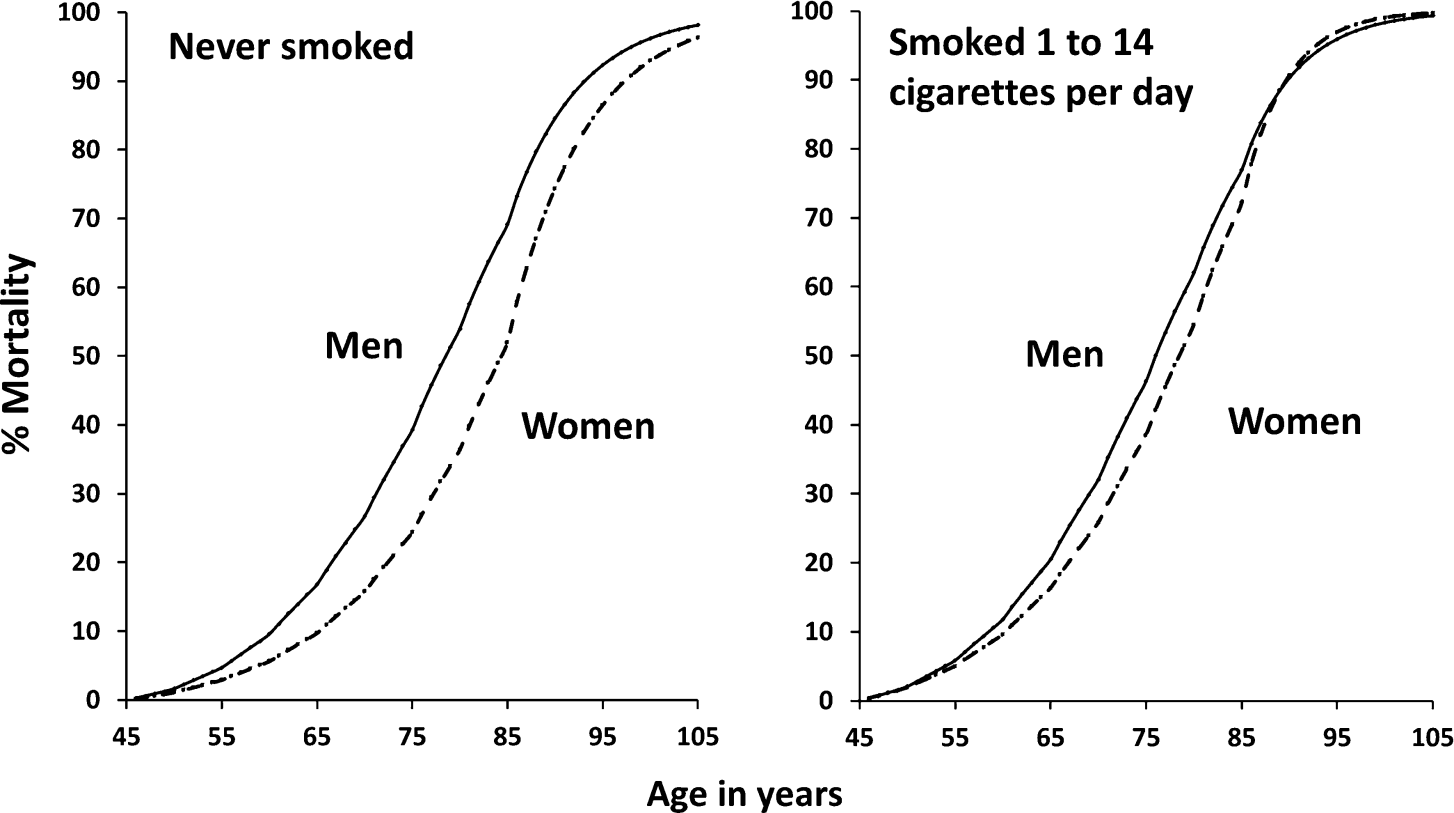
Predicted mortality curves, using a Weibull model for relative survival times, for men and women who attained age 45 at 1 January 1965 and who never smoked or who smoked 1–14 cigarettes per day (light smoking). *Solid lines* show mortality for men and broken lines for women

The fully-parametric models included a constant term and so the predicted percentiles of the survival distribution were computed. The percentiles were back transformed using the reference population hazards in order to build mortality curves as a function of age. This was especially easy to execute since a parametric survival model had been fitted and demonstrated the advantage of the reference relative survival time followed by parametric survival modelling. Figure 2 shows an example of this application; the effect of the higher risk to women of light smoking can be visualised by the narrowing of the gender gap compared to non-smoking. Therefore the reference relative survival approach can satisfy the aim of obtaining survival curves as a function of age.

The exponential model shown in Table 3 is identical to the model that would have been fitted had Poisson regression for IPD data been used [17, 18] and shows that the reference relative survival time-scale provides an alternative route for carrying out Poisson regression for IPD. The parameter values and their interpretation for the exponential model are very similar to those for the Weibull. Practitioners who are more comfortable with traditional methods can use the reference relative time scale and be confident that their results concur with Poisson regression and that the results will be similar to analysis using chronological age.

### Simulation study

For the values of the parameter β = 1.20 and β = 1.40, the estimated power was very similar when the simulated lifetimes were analysed using chronological age at risk as time-scale or the reference relative time-scale (Table 4; appropriate reference population). For both methods, when β = 1.00, the estimated probability of rejecting the null hypothesis of log(β) = 0 was similar to the type 1 error rate of 5 %. In all cases the mean values of the estimates of β were close to the true values and both methods showed no evidence of bias in estimation.

**Table 4:** Simulation results comparing the estimation of the parameter β using the reference relative time scale and the age at risk time scale using 200 simulations

	Chronological age as time-scale stratification by birth year and gender			Reference relative time as time-scale		
	Cox model			Cox model		
True value of β	1.000	1.200	1.400	1.000	1.200	1.400
Appropriate reference population						
Mean estimate of β	1.005	1.204	1.421	1.006	1.204	1.421
95 % CI lower bound	0.991	1.187	1.402	0.992	1.187	1.402
95 % CI upper bound	1.019	1.221	1.440	1.021	1.221	1.440
Estimated power (%)	6.5	42.5	93.5	5.5	44.5	92.5
95 % CI lower bound (%)	3.5	35.6	89.1	2.8	37.5	87.9
95 % CI upper bound (%)	10.9	49.7	96.5	9.6	51.7	95.7
Inappropriate reference population						
Mean estimate of β	1.005	1.200	1.399	1.003	1.198	1.398
95 % CI lower bound	0.992	1.185	1.380	0.990	1.183	1.379
95 % CI upper bound	1.018	1.216	1.418	1.018	1.213	1.417
Estimated power (%)	4.5	41.1	92.0	4.5	42.5	92.5
95 % CI lower bound (%)	2.1	34.1	87.3	2.1	35.6	87.9
95 % CI upper bound (%)	8.4	48.2	95.4	8.4	49.7	95.7

When lifetimes were simulated somewhat shorter than in the reference population, the reference relative time-scale method performed well and performed similarly to the chronological age at risk time-scale in terms of power and bias (Table 4; inappropriate population). The simulations thus supported the assertion that the reference relative time-scale was a viable alternative to using chronological age at risk.

## Discussion

Transforming data, to achieve conformity to a parametric statistical distribution, is a cornerstone of much data analysis and yet surprisingly it is not often used for survival data from cohort studies. It has been used frequently in reliability and performance theory where there is motivation to determine suitable usage or exposure measures which transform real time to new scales. The term ‘operational time’ has been used for a time-scale obtained by integrating the hazard function of an inhomogeneous process [26] and the expression ‘operational failure time’ has been used in reliability analysis to describe a transformed failure time. The transformation to the reference relative time-scale described here is a transformation to an operational failure time-scale which acts to remove a large component of the inhomogeneity in the observed survival times by using a reference population containing external knowledge of past temporal and cross-sectional hazard rates as a measure of this inhomogeneity. It need not be assumed that the reference population hazard rates apply directly to the cohort participants. Indeed the same rates would not apply if the cohort were not healthy individuals but were a group with a disease diagnosis. Rather it would be enough if the hazards rates could be assumed proportional for the removal of homogeneity to succeed. The transformation to the reference relative time-scale is a pre-processing of the data so that modelling can go ahead without concern for complex sub-models for study group demographics, such as age, or large numbers of strata in a stratified model which has concerned data analysts [5]. The modelling effort can focus on the risk factors of interest and parsimonious models can be determined. If there was concern that the pre-processing had not been totally effective, then additional covariates such as age and age squared could be included in the regression model. This would provide a test of lack-of-fit since the coefficients for these terms should be negligible if the pre-processing has achieved its objective. The pre-processing also makes it more likely that a suitable parametric survival model can be identified and this may provide insight into the underlying stochastic mechanisms.

In contrast, it can be argued that a primary objective of epidemiology is to understand the complex pattern of risk over age and over calendar time and that the removal of all or a part of this pattern may hinder rather than help meet this objective. If age and calendar time are no longer in the regression model then the pattern with age and calendar time cannot be visualised in the model coefficients. However, comparative patterns with age and calendar time can be obtained using a back transformation of the reference population as has been shown here but this requires additional effort beyond interpreting the regression coefficients. Also, if age and calendar time are included in the regression model fitted after pre-processing then a good deal of care would be needed in their interpretation. It is clear that transformation to the reference relative time-scale will not be appropriate in all applications and this is a limitation of the methodology.

A further limitation of the methodology as described here is that no account has been taken of the precision of estimation of the reference population hazard function. In the illustrative example actual mortality in England and Wales was likely to be reasonably well estimated but population size would have a greater degree of imprecision and this would hold true for many other potential reference populations. As described here, the methodology assumes that the reference population hazard is measured without error and, if this was in doubt, a sensitivity analysis would be needed to confirm study findings.

In the illustrative example given here, all covariates and factors were determined at baseline when participants entered the study. If covariates and factors varied during follow-up then the analysis would follow the usual method for handling time-varying covariates. The total calendar time interval on study for each individual would need to be sub-divided into consecutive time windows during which the covariates and factors were assumed constant. Each portion before the last would be censored and the final time window would reflect whether the participant finally had the event of interest or was censored at exit from the study. Then each time window would be separately transformed to the reference relative time scale and the regression analysis proceed as usual.

To reiterate, in reliability theory a definition of a ‘good time-scale’ has been proposed [10] with four defining features; (1) scientific relevance (2) parsimonious and accurate description of variation in failure times under different conditions (3) a compact statistical distribution on the transformed time-scale and (4) succinct and meaningful summarization of the effects of covariates of interest. The reference relative time-scale demonstrated these attributes in the illustration given here. The time-scale had several relevant interpretations, values on the scale could be defined for the widely differing individual participants, the Weibull provides a compact distribution on the new time-scale and effects of risk factors of interest were succinctly summarised by the model coefficients.

The reference relative time-scale discussed here is a transformation of the measure of individual relative survival that has been suggested previously in the literature [19]. Subsequently, the concept of relative survival seems to have evolved and relative survival is often used now to mean only the estimation of the excess mortality due to a particular disease or condition within an additive hazards model framework [27–30]. Since the disease or condition is likely to increase mortality not only from the specific condition or disease but also from other causes, all-cause mortality provides an easier quantity to measure than cause specific mortality. The reference population is used as a baseline from which to estimate the excess numbers of deaths. In this context, the models fitted are additive hazards models but the concept of relative mortality is equally applicable to proportional hazards models [10]. Where cause specific mortalities are available for the reference population, a reference relative time-scale can be defined for any specific cause and so relative survival, in the context described here, can be applied to both cause specific and all-cause mortality. Hence, there may be applications to the modelling of competing risks since a value on each of a number of reference relative time-scales for different causes of death could be computed to provide a vector of multivariate survival data in reference relative time. Further, the meta-analysis of IPD is of increasing importance [31]. The use of the reference relative time-scale may have benefit for meta-analysis of IPD since cohorts from different locations could each be transformed using different reference populations and then combined in a single regression analysis provided, of course, the same covariates were recorded in each location.

The reference relative time scale could be viewed as a composite time-scale which merges an age time-scale with a calendar time scale. Age–period-cohort (APC) models, which index an event count by age, calendar period and birth cohort, have an extensive literature [32] and have been of interest because of the recognised failure of identifiability of effects due to co-linearity. The reference relative time scale may have applications in APC modelling since it reduces the time scales from three to two and so models might become identifiable.

The use of a reference population to gain insight into the risk factors affecting a study cohort begs the question of whether results from the study group can be generalised to the reference population. This would seem likely if it could be assumed that the study group is, in some sense, a sample from the reference population, but might the generalizability be conditional upon certain other assumptions? It would be reasonable to use a reference population to create a relative time-scale so long as there was a sound belief that the hazards in the study group were proportional to those in the reference population. If this was the case, would generalisation to the reference population be valid also?

It may be worthwhile considering whether the definition of the reference relative time-scale as the integral over a reference population hazard could be extended to include an additional term under the integral for the quality of life of the individual as a function of age. The transformation to the reference relative time-scale stretches time when the population hazard is high and this effect is greater for older compared to younger individuals. A quality of life function could act as a penalty to reduce some of the stretching and would change conclusions regarding risk factors of interest when regression models are fitted on the reference relative time-scale. It is clear, therefore, from these discussions that the reference relative time-scale not only provides a viable alternative to modelling chronological age or real time-on-study but is an interesting concept in its own right that points the way to several avenues of future research which others may wish to explore.

## Conclusions

The reference relative time-scale was shown to provide a viable alternative to the current standard method which uses chronological age as the time scale with left truncation for age at entry and a Cox model stratified on birth cohort. Its use led to a simplification of the modelling process and the scale possessed the defined features of a good time-scale as defined in reliability theory. Simulation suggested that the two methods have similar power and are equally unbiased. The reference relative time-scale has several interpretations and provides a unifying concept that links contemporary approaches in survival and reliability analysis to the traditional epidemiologic methods of Poisson regression and SMRs and can be understood as an extension of these traditional methods. The community of practitioners has previously failed to make this connection.
